# Evidence of an Alternative Currency for Altruism in Laboratory-Based Experiments

**DOI:** 10.5964/ejop.v11i1.855

**Published:** 2015-02-27

**Authors:** Daniel Farrelly, Emma Moan, Kristi White, Sarah Young

**Affiliations:** a Institute of Health and Society, University of Worcester, Worcester, United Kingdom; b Department of Psychology, University of Sunderland, Sunderland, United Kingdom; University College Cork, Cork, Ireland

**Keywords:** altruism, cooperation, punishment, empathy, experimental

## Abstract

Research shows that altruistic behaviours arise in varying social situations in line with different theories of causes of such behaviours. However most research uses financial costs only, which makes our understanding of altruism currently limited. This study presents findings of three experiments that use a novel and simple laboratory-based task that measures altruism based on the amount of time participants are willing to spend as a cost to help others. This task assessed two specific theories; altruistic punishment (Experiments 1 & 2) and empathy-altruism (Experiment 3). All experiments showed that the task was successful, as participants were more likely to altruistically punish violators of social contracts than other scenarios (Experiments 1 and 2), and also incur more costs to behave altruistically towards others when feeling empathic than different emotional states (Experiment 3). These results provide clear support for the use and value of this novel task in future research.

## Introduction

Social behaviours such as cooperation or altruism in humans have been hard to explain, a fact that Darwin (1859) recognised when he stated that its existence was problematic to his theory of natural selection. Since this time however, different theoretical explanations of how altruistic^i^ behaviours could have evolved have been developed, such as kin selection (Hamilton, 1964a,b), reciprocal altruism (Trivers, 1971), indirect reciprocity (Nowak & Sigmund, 1998), competitive altruism (Roberts, 1998), costly signalling (Gintis, Smith, & Bowles, 2001) and altruistic punishment (Fehr & Gächter, 2002). These theories identify the social conditions under which altruism can be an adaptive behaviour in humans, and explanations such as the empathy-altruism hypothesis (Batson et al., 1991) offer proximate mechanisms by which it arises.

Importantly, experimental research with humans has generally supported the above theories and thus helped our understanding of altruistic behaviour (e.g. Burnstein, Crandall, & Kitayama, 1994; Farrelly, Lazarus, & Roberts, 2007; Fehr & Gächter, 2002; Gurven, 2004; Sylwester & Roberts, 2010; Wedekind & Milinski, 2000). However the vast majority of such studies rely on using economic games such as the Prisoners Dilemma and Public Goods Games, with a willingness (or lack of) to incur financial costs being the main currency with which altruism is revealed. This narrow view of altruism ignores the fact that costs can be in different currencies and as a result most of the support for the above theories can only really conclude that such theories can explain financial altruism.

Therefore further examination of alternative types of costs in altruism is necessary for a fuller and detailed understanding. This will be particularly true for costs high in ecological validity, such as time and energy, which would have universal and equivalent understanding across all populations. Previous research within the experimental social psychology literature have attempted to use measures of intention to help in a number of different ways, such as through willingness to volunteer or theoretically offering help (Garcia, Weaver, Moskowitz, & Darley, 2002; Nelson & Norton, 2005) or number of charity pamphlets taken to potentially redistribute (Pichon, Boccato, & Saroglou, 2007). However there is a difference between the intention to help and actually incurring costs to help, which is particularly pertinent from an evolutionary perspective where the latter is the only reliable indicator of altruistic behaviour. Some studies have successfully managed to assess real-life altruistic behaviour such as likelihood of helping others pick up dropped pens (Macrae & Johnston, 1998) or books (Scaffidi Abbate, Ruggieri, & Boca, 2013), using painful exercises (Madsen et al., 2007), responses to email requests for help (Oates & Wilson, 2002), and hunting in different cultures (e.g. Smith, Bird, & Bird, 2003). These novel approaches however can be difficult to replicate in more controlled laboratory-based experiments (because of issues such as costs, ethical content, and facilities). Therefore there is a need to develop tasks that can be reliably used in future experimental psychology research to measure how willing a participant is to incur a cost, other than a financial one, to directly benefit another.

As a result, we present here the results of three experiments that utilised such a task. These experiments used the task to see if participants’ altruistic behaviour supported predictions from altruistic punishment (Experiments 1 and 2) and empathy-altruism (Experiment 3). Below we provide further details of each experiment separately, and also of the altruistic task that each used.

## Experiment 1

Fehr and Gächter (2002) have shown that when given the ability, humans will incur a cost to punish another individual who has cheated in a public goods game. As a result, they suggest that the presence of ‘altruistic punishers’ in populations can increase overall levels of altruism in different social groups. Furthermore Fehr and Gächter (2002) suggest that individuals are motivated to altruistically punish others due to negative emotions, as participants presented with imaginary scenarios of the game felt more anger towards imaginary cheaters than imaginary cooperators. According to Fehr and Gächter (2002), this emotional response is the proximate mechanism that leads to individuals willing to incur costs to punish cheaters. This is even true if they will not interact with the cheater in the future, and therefore would not directly benefit from the reforming power of the punishment, which can turn cheats into cooperators.

As a result, this experiment aimed to see if the motivation individuals have to altruistically punish cheats extends to them incurring costs other than financial to punish in imagined scenarios. This was explored using a novel, non-financial altruistic task, to also examine its validity as a measure of altruistic behaviour in an experimental setting. In this task, hypothetical scenarios were presented to participants, and they were able to respond to this to affect the scenario’s outcome. The level of their response varied according to how much time and effort they were willing to perform^ii^. As a result, it was predicted here that individuals would be more altruistic (i.e. would incur more costs through increased time and effort) to punish individuals in scenarios who had violated social contracts than when they violated in more neutral ways.

### Methods

#### Participants

Sixty-eight participants took part in this experiment. These participants and those in subsequent experiments were mainly undergraduate students who participated for course credit, and all experiments received ethical approval from the university’s ethics committee.

#### Materials

**The Altruistic Task.** This was a pen and paper task, where participants were presented with six scenarios that described a particular event where a negative outcome had occurred. Three of these scenarios involved a violation of a social contract by an individual (e.g. “You see a person being pulled over by the police for using a mobile phone whilst driving”), and the other three did not (e.g. “You go to your local cinema and the details advertised about the films are incorrect”). After each scenario, participants were able to decide which actions they would like to happen in response to the scenario (e.g. the size of fine they driver received, or the length of time before returning to the cinema). Each action varied on a ten-point scale (e.g. £0 - £90 in £10 increments for the fine, and 0 – 9 months in 1 month increments before returning to the cinema), and participants could select which point (i.e. the size of the punishment) they wished to occur for each scenario. Table 1 summarises the different scenarios and responses used in this experiment.

**Table 1:** Details of the Different Scenarios and Their Response Used in Experiment 1

Scenario	Response
Social Contracts	
Observing a waiter being verbally abused by another customer in a restaurant	Length of time before the waiter serves this customer again (0 minutes – 9 minutes, 1 minute increments)
See driver pulled over by the police for using mobile phone	Size of fine driver receives (£0 - £90, £10 increments)
See individual pushing in front of queue to use cash machine	Number of places individual is put back in queue (0 – 9 places, 1 place increments)
Neutral	
Local cinema advertising incorrect details about film times	Length of time before returning to cinema (0 – 9 months. 1 month increments)
Landline telephone broken due to bad weather	Length of time willing to stay with telephone company (9 – 0 months, 1 month increments)
Item of clothing incorrectly priced in sale, although shop accept labelled sale price	Length of time before returning to shop (0 – 9 months, 1 month increments)

In order for the size of response to be altruistic (i.e. incur a cost), participants were required to complete a short puzzle for each point on the scale up to their chosen response. These puzzles were simple but time-consuming, and were in six categories; writing numbers in word form (e.g. 39,444), listing countries beginning with a particular letter (e.g. C), making six-letter words using letters from a random string of letters, listing words with ‘oo’ in them, solving six-letter anagrams (e.g. CUREES), and mental arithmetic (e.g. 6+[8x9]). Therefore, if for example in the driver/mobile phone scenario a participant wished the driver to be fined £40, they would need to incur the costs of completing five puzzles, as £40 is the fifth point on the scale of £0 - £90. As a result, this task represents a reliable measure of altruistic behaviour as it is positively correlated to the amount of cost an individual is willing to incur (in terms of increased time completing the task) to punish. The total number of points (in other words, total number of puzzles completed) in the three scenarios for each condition (Social Contract, Neutral) were then calculated.

#### Procedure

Participants completed all parts of this experiment in a private cubicle. After information was presented and consent obtained, participants were told about the altruistic task, and presented with a brief example. They were also asked by the experimenter if they had any questions about the task, to ensure that they fully understood. They then went through the six different scenarios (the order of these was randomly allocated) and circled the point on each scale that was their response to the scenario.

After this, participants were presented with a review of their decisions (which was completed by the experimenter), and then were informed of the total number of puzzles they needed to complete (this could be anywhere in the range of 0 – 60). Participants were then free to select any of the puzzles from the six categories (see The Altruistic Task section for more details) to reach their total. Once the correct number of tasks was completed^iii^, the participant was debriefed and the experiment completed.

### Results and Discussion

To assess the difference in amount of altruistic punishment between the two scenario types (Social Contract, Neutral), a paired samples *t*-test was conducted. This revealed a highly significant difference, *t*(67) = 12.4, *p* < .001, *r* = .83, with Social Contract scenarios (*M* = 15.31, *SD* = 5.96) being punished more that Neutral scenarios (*M* = 4.29, *SD* = 4.71).

These results provide support for the prediction. Overall participants did significantly vary the costs they were willing to incur in the tasks, and incurred more costs to punish violators of social contracts over violators of more neutral scenarios. This therefore offers very strong support for the use of the tasks in experimental altruistic research, as it shows that participants’ motivation to incur costs to punish others extends to hypothetical scenarios and beyond financial costs, and is also commensurate with predictions from altruistic punishment (Fehr & Gächter, 2002).

## Experiment 2

Although successful in showing participants respond differently in relation to the altruistic nature of the scenario task as predicted, the similarities between the social contract and neutral scenarios in Experiment 1 were quite marked. Would the large difference in altruistic punishment participants were willing to engage in remain when the scenarios were more similar? This we aimed to investigate in Experiment 2, by varying the intention behind the violation of social contracts. In other words, different versions of the scenarios were again used in this experiment, and all were based on social contracts. The only difference here though was that violations of these contracts were either done intentionally or non-intentionally. As altruistic punishment is believed to be conducted with the aim of preventing cheats from cheating again (Fehr & Gächter, 2002), it was predicted here that individuals would incur more costs punishing intentional violators of social contract imaginary scenarios than non-intentional violators.

Furthermore, this experiment investigated differences in how much individuals respond to the emotions evoked by cheating behaviour with altruistic behaviour in this task In particular, this experiment explored how much participants’ abilities to regulate and interpret emotions could play an important role. Therefore Experiment 2 also took into account individuals’ emotional intelligence (EI) levels. In this burgeoning area of research, measures of emotional intelligence such as that of Schutte et al. (1998) are based on the model of EI put forward by Salovey and Mayer (1990), which is partly based on the abilities we have to regulate and utilise emotions in everyday life. This will have important implications for how participants deal with the emotional response to the hypothetical scenarios, but how it will affect the punishment of violations of social contracts is debatable. On one hand, it could be predicted that EI will positively correlate with how much altruistic punishment an individual is willing to incur as this is a strategy to regulate emotions as Fehr and Gächter (2002) may predict (in other words, do individuals use the opportunity to punish cheaters to relieve the anger they feel?). However, this may not necessarily be the case as individuals high in EI may not need the opportunity to punish to regulate the negative emotions they are feeling, as they have other more successful strategies available. Therefore the measure of EI will be used in an exploratory manner in Experiment 2 to investigate how/if it relates to altruistic punishment in the scenarios task, and to further explore the reliability of the scenario task to elicit altruistic behaviour experimentally.

### Methods

#### Participants

Sixty-three participants took part in this experiment.

#### Materials

**The Altruistic Task.** This had the same layout as the task used in Experiment 1. Here though, three scenarios involved a violation of a social contract by a particular individual that was intentional, and the other three were violations of a social contract that were non-intentional (see Table 2 for a summary of these scenarios, and the responses). Another difference of the task in this experiment was that the scenarios and the puzzles to act as responses to them were included together and separately from other scenarios, so that participants would read the scenario and then need to make their response straight afterwards. Additionally, for each scenario there was only one category of puzzle that participants could select from, and these were either mental arithmetic or anagram puzzles. For each scenario, participants had a total of eighteen puzzles from which they could freely select.

**Table 2:** Details of the Different Scenarios and Their Response Used in Experiment 2

Scenario	Response
Intentional	
Fellow student does not bring work to group sessions.	Percentage deduction from their grade (0%– 50%, 5% increments)
Company purposefully not paying enough tax	Size of fine company receives (£0 - £50000, £5000 increments)
See individual pushing in front of queue to use cash machine	Number of places individual is put back in queue (0 – 10 places, 1 place increments)
Non-intentional	
Fellow student forgets to bring work to one group session.	Percentage deduction from their grade (0%– 50%, 5% increments)
Self-employed businessman accidentally fills out tax forms incorrectly	Size of fine businessman receives (£0 – £2000, £200 increments)
See individual in rush to a job interview forgets to buy train ticket	Size of fine this individual receives (£0 - £20, £2 increments)

**Emotional Intelligence Measure**. The Schutte Emotional Intelligence Scale (SEIS; Schutte et al., 1998) was administered to participants in pen and paper form. This is a 33-item self-report scale used to assess trait EI, and incorporates the following facets of EI: regulating emotions, regulating other’s emotions, perceiving emotions and utilising emotions.

#### Procedure

This was the same as for Experiment 1. After participants had completed all the scenarios and made their responses, they then completed the SEIS.

### Results and Discussion

Again, to assess the difference in amount of altruistic punishment between the two scenario types (Intentional, Non-intentional), a paired samples *t*-test was conducted. This again revealed a highly significant difference, *t*(62) = 12.1, *p* < .001, *r* = .84, with Intentional scenarios (*M* = 15.14, *SD* = 7.06) being punished more that Non-intentional scenarios (*M* = 4.25, *SD* = 4.31). To assess the possible role of EI in punishing behaviour, Pearson correlations were conducted between participants’ EI score and their levels of punishing. It was found that there was no significant correlation between EI and punishment levels for either intentional, *r*(63) = -.001, *p* = .99, and non-intentional scenarios, *r*(63) = -.14, *p* = .26.

These results again provide support for the predictions. Participants did incur greater costs to punish intentional violators of social contracts than non-intentional violators. However from the exploratory analysis, it was found that emotional intelligence had no significant influence on levels of altruistic punishment in this experiment.

Overall the results of these first two experiments clearly show that in a laboratory-based task that is not financially based and uses imaginary, hypothetical scenarios, individuals do exhibit very strong altruistic tendencies in social situations that fit the predictions of altruistic punishment (Fehr & Gächter, 2002). The final experiment included here looked at how the altruistic task used above can also be used to measure altruistic behaviour per se, using emotion priming.

## Experiment 3

The aim in this final experiment was to explore whether the parameters of the task used in Experiments 1 and 2 could also be used to directly measure an individual’s altruistic behaviour. In other words, could this laboratory-based task that uses non-financial costs be reliably used to show how willing an individual is to incur costs to help another? It was predicted here that this would be so. Also, in response to the non-significant findings in relation to emotional intelligence in Experiment 2, this experiment sought to examine if an alternative individual difference measure, that of an ‘altruistic personality’ (Rushton, Chrisjohn, & Fekken, 1981), would be more suitable in accounting for behaviour in this version of the altruistic task, and also test the validity of the task as a measure of altruism.

Furthermore, this experiment examined whether inducing different emotions in individuals by means of priming would affect altruistic tendencies. This experiment aimed to do this by presenting different videos to participants to induce emotions. Whereas anger is considered to be a necessary emotion to drive altruistic punishment, this will not be the case for actual altruism. Therefore the choice of emotions was based on the empathy-altruism hypothesis (Batson et al., 1991), which suggests that empathic concern is a necessary social-psychological state for altruism to occur, as individuals who feel this concern are motivated to incur costs to help others in need. Therefore ‘empathy’ was an emotion/mood that was primed in this experiment, and this was compared to ‘anger’ and ‘neutral’ primes.

It was therefore predicted that participants primed to feel empathy will incur more costs to be altruistic in the task than those primed to feel either anger or a neutral state. Furthermore, it was predicted that altruistic personality (as measured by the SRA) would positively predict individuals’ responses in the altruistic task.

### Methods

#### Participants

Seventy-two participants took part in this experiment.

#### Materials

**The Altruistic Task.** This followed the same format as in Experiments 1 and 2, in that it involved the presentation of scenarios which were followed by a scale of responses that could be completed following incurring the cost of solving puzzles. However in this experiment these scenarios involved an opportunity for the participant to behave altruistically (e.g. sharing chocolates with work colleagues), and their response would positively correlate with how altruistic they were (e.g. complete more puzzles in order to share more chocolates). In total there were eight scenarios, see Table 3 for a summary of these. As with Experiment 2, a category of puzzle was linked directly to each scenario, and details of these are also included in Table 3.

**Table 3:** Details of the Different Scenarios and Their Responses, as Well as the Puzzle Category for Each Scenario Used in Experiment 3

Scenario	Response	Puzzle category
Help lady in a hurry move up a queue	Number of places lady is moved forward in queue (0 – 9 places, 1 place increments)	Anagrams
Child unable to afford new bus ticket price	Amount of money to give to child (£0 - £2.00, £0.2 increments)	Mental arithmetic
Sharing days of holiday (won in competition) with work colleagues	Number of days shared (0 – 10 days, 1 day increments)	List different birds
Tourists in shopping centre struggling to get through door	Number of tourists door held open for (0– 10 tourists, 1 tourist increments)	Anagrams
Schoolchildren unable to go on trip as bus has broken down	Number of children that participant can transfer (0 – 9 children, 1 child increments)	Combine letter strings to make words (e.g. SCO – OTER)
Stray dog in need of veterinary care	Percentage of vets bill paid (0% - 100%, 10% increments)	Anagrams
Sharing chocolates with work colleagues	Number of chocolates shared (0 – 10 chocolates, 1 chocolate increments)	List different plants
Local community centre needs help running activities	Number of days helping out (0 – 10 days, 1 day increments)	Mental arithmetic

**Emotional Prime – Videos.** Brief videos (between 2 and 3.5 minutes) that are freely available on the internet were presented to participants. There were three emotion conditions (Anger, Empathy, Neutral) that participants were randomly assigned to, and there was one video included with each condition to prime these. Each of the videos were on a similar subject (dog care/ownership), but the content varied according to the emotion they were priming; the video in the anger condition was about a dog that had been wrongly shot and killed by police (www.youtube.com/watch?v=bIAbnLKNVAo), the video in the empathy condition was about an abandoned dog that had been cared for and recovered in an animal shelter (www.youtube.com/watch?v=OOZVkSRQ8CU), and the video in the neutral condition was about how to groom and brush a dog (www.youtube.com/watch?v=Jb8PKrqtWI8).

**Self-Report Scale (SRA).** Participants completed the Self-Reported Altruism Scale (Rushton et al., 1981). This is a 20 item self-report measure that includes a list of altruistic behaviours, such as “I have given money to a charity” or “I have donated blood”, and asks participants to respond on a 5-point scale from “Never” to “Very often.”

#### Procedure

To begin with, participants completed the SRA online and then contacted the experimenter to arrange a later session to complete the remainder of the experiment in a laboratory. In this later session, participants were randomly allocated to one of the three emotion conditions and then were requested to watch the corresponding video in a private booth. After watching the video, participants then went on to complete the altruistic task.

### Results and Discussion

A univariate ANOVA was conducted with emotion condition (Empathy, Angry, Neutral) as a between subjects factor on total amount of altruistic responses in the task. There was found to be a highly significant main effect of condition, *F*(1,69) = 36.62, *p* < .001, µ^2^ = .52. Subsequent pairwise comparisons (Bonferroni) found that participants primed with empathy made significantly more altruistic responses (*M* = 66.58, *SD* = 10.74) than those primed with anger (*M* = 34.63, *SD* = 14, *p* < .001, *r* = .8) and those primed with a neutral video (*M* = 44.54, *SD* = 14.66, *p* < .001, *r* = .68). Also those primed with neutral videos made significantly more altruistic responses than those primed with anger (*p* = .035, *r* = .33).

Finally, a multiple regression analysis was conducted with SRA score entered at step 1 and emotion condition (using dummy variables) entered at step 2 as predictors, with total number of points of altruistic behaviour across all scenarios as the outcome variable. At step 1, there was a near significant positive effect of SRA score on altruistic responses, *t* = 1.97, *p* = .053. At step 2, it was found that being in the empathy condition positively affected altruistic responses, *t* = 5.59, *p* < .001, and being in the angry condition negatively affected altruistic responses, *t* = -2.57, *p* =.021. The final model was significant, *F*(1,71) = 24.07, *p* < .001, µ^2^ = .52, and explained 51.5% of the variance.

These results support the predictions. Firstly it was found that participants primed to feel empathy incurred more costs to be altruistic to hypothetical others in the laboratory-based task than those primed to feel either anger or no emotion. Furthermore overall altruistic behaviour in the task positively correlated with SRA, a measure of an altruistic personality, although not quite significantly. As a result, this experiment provides further support to the use of this novel laboratory-based task as a reliable measure of the costs an individual is willing to incur to help another.

## General Discussion

The aim of the experiments of this study were to examine whether the conditions of the altruistic task, outlined above in its different forms in the different experiments, could be used to reliably measure altruistic behaviours in a currency other than financial ones in a simple lab-based setting based on hypothetical scenarios. This aim was strongly supported throughout, as individuals were shown to behave more altruistically in response to these hypothetical scenarios, most importantly in manners that were explicitly predicted by different theories (altruistic punishment, empathy-altruism). Also, there is evidence that altruistic behaviour in this task positively relates to a standard measure of altruistic personality (SRA), further supporting its validity. As a result, there are clear reasons to believe that this task can be used to measure altruistic responses to social situations that would test other theories, such as altruism as a costly signal (Gintis et al., 2001) or kin selection (Hamilton, 1964a,b). Also of particular interest would be to examine potential sex differences in altruistic behavior, as although this was not assessed in the current experiments, previous research has highlighted the importance of analyzing this (e.g. Eagly & Crowley, 1986; Farrelly et al., 2007). Furthermore, the results of Experiment 3 show that altruistic behaviours in the task can be experimentally manipulated, in this case via the priming of different emotional states. This is a further quality of the task that has been revealed and presents a great deal of opportunities for future research. Overall this suggests that research in human altruistic decision-making can now go beyond the standard and near-ubiquitous use of economic financial games in its experimental designs.

A potential issue with the task used is the costs involved. The puzzles used were deliberately constructed to not be too demanding, as the aim was for them to be time-consuming rather than difficult. This meant that there was some compromise in the size of the cost they elicited, which could be argued means that they were not as costly as we suggest. However this does not explain why the simple puzzles were completed alongside different scenarios in patterns clearly predicted by the theories, rather than randomly. Also, there was the possibility of demand characteristics occurring, with some scenarios less subtle than others. However this was possibly only in a minority of scenarios across the three experiments, and furthermore participants were still willing to incur costs, suggesting that demand characteristics, if they did exist, would not be powerful enough to fully explain our significant findings.

A further consideration is the costs incurred in the task may not be subjectively equivalent across all participants. For example, a busy participant, whose time is very limited, will incur a greater cost when completing the same amount of puzzles as a less busy participant. However it is anticipated that such variation would be less of an issue than for costs in terms of finances, where variation could be much greater between participants (i.e. the difference between richest and poorest participant could theoretically be much greater than the difference between the busiest and least busy participant). Furthermore, the pattern of results observed across the three experiments suggests that any effect of different subjective costs of time did not adversely affect our findings. However this raises an interesting potential area for future research, as any manipulation of time constraints may provide more rigorous and tighter findings with regards to altruistic behavior, For example such experiments could directly manipulate the time available to participants as Macrae and Johnston (1998) did with their ‘running late’ study, or by conducting experiments at different periods when participants’ available time will vary in value (e.g. during lunch breaks as oppose to at the start of lectures).

### Conclusion

These experiments highlight a novel task that can be used to measure altruistic behaviour in a simple and cost-effective manner in future laboratory-based social psychology experiments. This can be beneficial for experimenters as it allows future research to expand our understanding of altruism beyond the rather restrictive costs in terms of mainly finances. Furthermore as shown here, the details and conditions of the task can be easily altered to assess different theories and different predictions. It is therefore our aim for future research to use versions of this task in novel ways in different circumstances to allow a richer and clearer understanding of why humans are altruistic.
